# Habitual lip and tongue posture in infants with Trisomy 21: characterization and associated factors

**DOI:** 10.1590/2317-1782/e20250157en

**Published:** 2026-06-19

**Authors:** Letícia Pereira Quintaes Godinho, Larissa Melgaço Campos, Andréa Rodrigues Motta, Renata Maria Moreira Moraes Furlan

**Affiliations:** 1 Universidade Federal de Minas Gerais – UFMG - Belo Horizonte (MG), Brasil.; 2 Programa de Pós-graduação em Ciências Fonoaudiológicas, Universidade Federal de Minas Gerais – UFMG - Belo Horizonte (MG), Brasil.; 3 Departamento de Fonoaudiologia, Universidade Federal de Minas Gerais – UFMG - Belo Horizonte (MG), Brasil.

**Keywords:** Down Syndrome, Muscle Hypotonia, Chromosomes, Human, Pair 21, Tongue, Lip

## Abstract

**Purpose:**

To characterize the habitual lip and tongue posture in infants with trisomy 21 and to verify its association with age, sex, and clinical characteristics.

**Methods:**

This is an observational, cross-sectional, analytical study with a sample of 61 infants with trisomy 21, with a mean age of 7.4 ± 6.2 months. The habitual lip and tongue posture was obtained through the analysis of videos of the infants' faces. Data regarding age, sex, and clinical conditions were collected from the participants' medical records. The study response variables were the habitual tongue and lip postures predominantly adopted by the infants. Explanatory variables included age, sex, prematurity, birth weight, heart disease, hypothyroidism, lung disease, breastfeeding, and use of bottles and pacifiers. Data were analyzed using descriptive statistics and Pearson's chi-square test for association between variables, with a 5% significance level. The study used 20% of the videos to verify the kappa coefficient and obtain intrarater and interrater agreement, classified as excellent and substantial, respectively.

**Results:**

The longest postures were parted lips (48.2%) and tongue inside the oral cavity (32.4%). Lip posture was associated with age at the time of assessment and birth weight, and tongue posture was associated with breastfeeding.

**Conclusion:**

Younger chronological age at the time of assessment and higher birth weight were associated with better lip posture, while breastfeeding was associated with better tongue posture.

## INTRODUCTION

Trisomy 21 (T21), also known as Down syndrome, is a genetic condition caused by the presence of an extra copy of chromosome 21 in all or some of an individual's cells^(1)^. This genetic alteration results in 47 chromosomes instead of the usual 46 and can occur through abnormal cell division at the time of fertilization^(2)^. T21 implies physical and cognitive characteristics, such as delayed motor and intellectual development and facial peculiarities, such as almond-shaped eyes, short neck, and muscle hypotonia^(1)^. The syndrome affects approximately one in 800 live births, being one of the most common genetic conditions^(2)^. Its incidence increases with advanced maternal age^(3)^.

Children with T21 often present speech-language-hearing disorders involving both orofacial motor skills and communication^(4,5)^. They may have significant feeding difficulties, including problems with breastfeeding^(6)^ and swallowing^(7)^. Furthermore, delayed language development is a common feature, as are difficulties in speech development and word articulation^(5)^. Anatomical alterations, such as macroglossia and a high and narrow palate, can aggravate these difficulties^(8)^.

Studies that evaluated the orofacial structures of infants with T21 found hypotonic lips and tongue, impacting the habitual posture of these structures. This suggests that the decrease in tone in the labial and mandibular muscles leads to difficulty in keeping the lips closed, resulting in parted or open lip postures^(4,9)^. In individuals with T21, muscle hypotonia generally affects all body musculature, being explained by structural and functional characteristics of the central nervous system, such as reduced volume and number of cells in the cerebral cortex and cerebellum and altered connectivity between neurons and other structures^(10)^.

Changes in habitual lip and tongue posture are related to the breathing pattern, with open lips and tongue on the floor of the mouth being observed in mouth-breathing children^(11)^. A study^(12)^ observed that tongue posture within the oral cavity and in a higher position in typical newborns was associated with a more symmetrical expiratory flow, suggesting that the relationship between breathing and tongue posture can be found in the first days of life. This reinforces the importance of focusing on the usual orofacial postures of infants, given the detrimental effects of mouth breathing on child development and quality of life^(13)^. Moreover, inadequate postures of these structures can negatively impact craniofacial development, with possible long-term consequences, such as maxillary underdevelopment and dental malocclusion^(8,14)^.

Studies address orofacial myofunctional changes in children with T21, pointing to inadequate lip and tongue postures^(4,9)^. However, no studies were found that investigated the factors associated with the adoption of these postures. It is still unclear what factors may influence the habitual tongue and lip posture in these children. Understanding these factors is essential, as it would allow for a more accurate diagnosis and a better-targeted approach to intervention to minimize the negative impacts of postural changes and, consequently, ensure healthy orofacial development.

Thus, this study aimed to characterize the habitual lip and tongue posture in infants with T21 and to verify the association with age, sex, and clinical characteristics.

## METHODS

This study adopted a quantitative approach and followed a cross-sectional, analytical, observational design. The study was approved by the Research Ethics Committee of the Federal University of Minas Gerais (UFMG) under approval 4.381.966, CAAE 37828920.1.0000.5149. All parents and/or guardians of the infants signed an informed consent form.

Data were collected at UFMG’s Dental School and involved 61 infants participating in an outreach program for infants with T21. All patients in the project who met the established criteria were included in the study. No sample size calculation was performed; the sample was determined based on the number of babies served, according to the outreach program’s database.

The study included infants with corrected gestational age between 6 months and 3 years, diagnosed with T21, and excluded Infants with other associated syndromes, orofacial malformations, or using enteral feeding tubes. The exclusion criteria were verified by analyzing the infants’ medical records in the outreach program.

The study had two stages to meet its objectives: collection of clinical data through analysis of the participants' medical records and access to video recordings of the infants' faces to observe their habitual lip and tongue posture.

### Clinical history

The study extracted from the medical records information on the infants’ age, sex, and clinical characteristics, including health history (associated syndromes, malformations, prematurity, birth weight, lung disease, heart disease, and hypothyroidism), breastfeeding, and use of feeding tubes, bottles, and pacifiers.

### Analysis of habitual lip and tongue posture

The habitual lip and tongue posture was verified by analyzing videos of the infants' faces, usually taken during the first consultation in the outreach program. The infants were recorded on their parents' laps, who were seated in a chair with a backrest. The videos lasted 5 minutes, focusing on the infant's face.

The videos were analyzed using Media Player Classic^®^ software, being paused second by second. In each second, the tongue posture was classified as: I) inside the oral cavity (tongue behind the lower gingival ridge or behind the lower incisor teeth); II) between the gingival ridges (tongue on the lower gingival ridge and behind the lower lip); III) on the lower lip (tongue touching the lower lip); IV) severe protrusion in relation to the lower lip (protruded tongue, on the lower lip, with the apex exceeding the anterior limit of the lower lip)^(4)^. Lip posture was classified as: I) closed (contact between the lower and upper lips along their entire length); II) parted (contact between the upper and lower lips only near the corner of the mouth); or III) open (no contact between the lower and upper lip)^(4)^. The seconds the infant remained in each classification of habitual lip and tongue posture were counted, with the posture adopted during most of the video time being considered predominant, compared to the other isolated postures. The analysis did not consider moments when the infant smiled or vocalized. One researcher analyzed all videos and, to increase data reliability, a second researcher independently analyzed 20% of the videos, verifying interrater agreement. The first researcher also reanalyzed 20% of the videos 6 months after the first analysis, verifying intrarater agreement. The kappa coefficient was calculated to verify interrater and intrarater agreement^(15)^.

### Data analysis method

The study response variables were habitual lip and tongue posture predominantly adopted by infants, while the explanatory variables were sex, age, prematurity, birth weight, lung disease, heart disease, hypothyroidism, breastfeeding, bottle feeding, and pacifier use.

Descriptive statistical analysis was performed, presenting measures of central tendency and dispersion for the numerical variables (percentage of time in lip and tongue postures and age at the time of assessment) and absolute and relative frequencies of categorical variables (sex, prematurity, lung disease, heart disease, hypothyroidism, breastfeeding, bottle feeding, and pacifier use). The numerical variables age at the time of assessment and birth weight were transformed into categorical variables, considering the cutoffs of 6 months for age at the time of assessment and 2,500 g for birth weight (low weight: below 2,500 g; adequate: equal to or above 2,500 g)^(16)^.

Inferential statistical analysis was performed using Pearson's chi-square test for association between variables. All analyses considered a 5% significance level and used Stata 13.0 software.

The effect size was calculated, being considered insignificant when less than 0.19, small between 0.20 and 0.49, medium ​​between 0.50 and 0.79, and large when greater than 0.80^(17)^.

Interrater and intrarater comparison was performed for agreement analysis (Table 1). Interrater agreement was rated as substantial for both lips and tongue, while intrarater agreement was excellent for both postures, according to the criteria of Landis and Koch^(15)^.

**Table 1:** Results of interrater and intrarater agreement

Variable	Interrater agreement		Intrarater agreement	
	kappa	Classification	kappa	Classification
Habitual lip posture	0.7477	Substantial	0.815	Excellent
Habitual tongue posture	0.7544	Substantial	0.900	Excellent

Kappa test

Caption: Classification: weak (0 to 0.2); fair (0.21 to 0.40); moderate (0.41 to 0.60); substantial (0.61 to 0.80); and excellent (0.81 to 1.0)^(15)^

## RESULTS

The study included 61 infants, with a mean age of 7.4 months at the time of assessment (SD = 6.2 months, minimum of 1 month and maximum of 26 months); 24 were female (39.3%), and 37 were male (60.7%).

**Figure 1:**
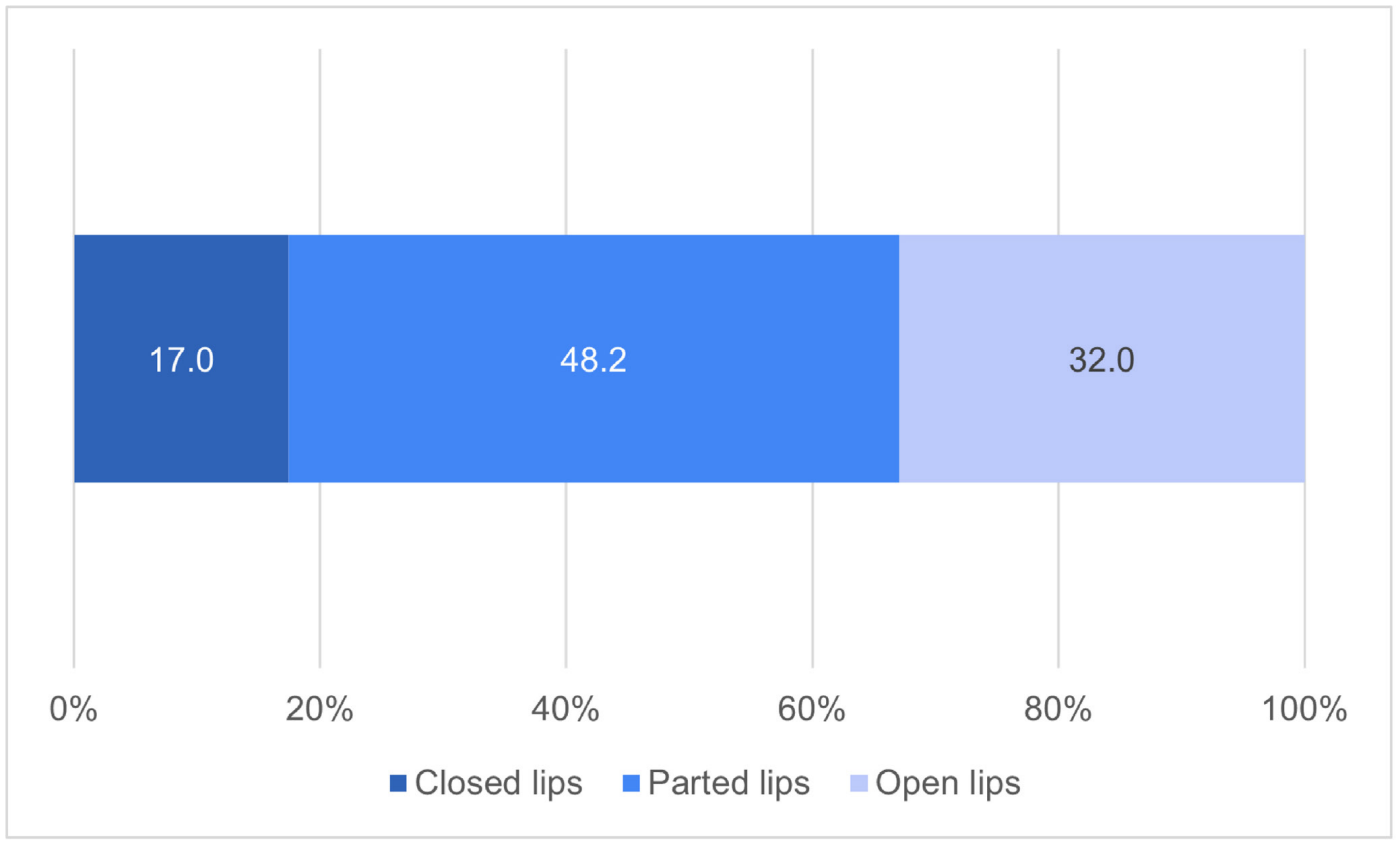
Duration of each typical lip posture as a percentage of the total video time

**Figure 2:**
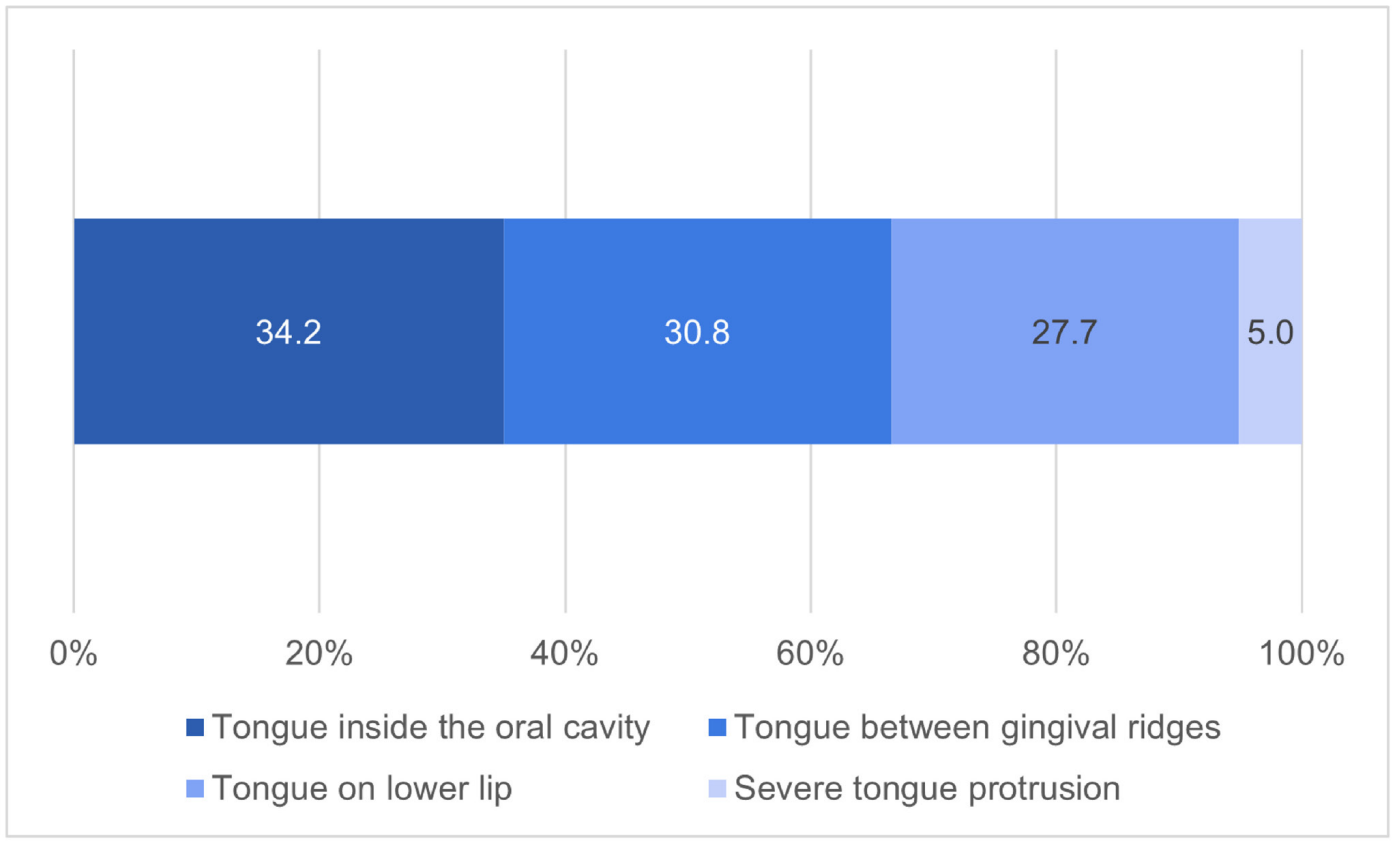
Duration of each typical tongue posture as a percentage of the total video time

Figures 1 and 2 show the average amount of time that the infants assumed each of the usual lip and tongue postures, respectively, presented as a percentage of the maximum video duration. The postures adopted for the longest time in the videos were parted lips and tongue inside the oral cavity. Combined inappropriate tongue postures accounted for 63.5% of the video time, and lip postures for 80.2%.

Regarding the number of individuals classified according to their predominant habitual posture, there was a higher frequency of individuals with a predominance of parted lips (n = 39, 63.9%), followed by open lips (n = 18, 29.6%) and closed lips (n = 4, 6.5%). Also, there was a greater number of individuals classified with a predominant tongue posture between the gingival ridges (n = 22, 36.1%), followed by tongue inside the oral cavity (n = 21, 34.4%) and on the lower lip (n = 18, 29.5%). No participant presented severe protrusion as the predominant tongue posture.

Table 2 presents the association analysis between habitual lip posture and explanatory clinical variables. Lip posture was associated with birth weight; a greater number of participants who were born with adequate weight predominantly presented parted lips, and a greater number of infants who were born with low weight predominantly presented open lips. Lip posture was also associated with age at the time of assessment; none of the participants assessed after 6 months presented closed lips as the predominant posture. The effect size was small for both associations.

**Table 2:** Association analysis between habitual lip posture and the explanatory clinical variables

Variable	Habitual lip posture (n = 61)						p-value^A^	Effect size^B^
	Closed lips		Parted lips		Open lips			
	n	%	n	%	n	%		
Sex							0.822	0.0802
Females	1	4.2	16	66.7	7	29.2		
Males	3	8.1	23	62.2	11	29.7		
Age							**0.043** ^*^	0.3207
Up to 6 months	4	10.5	20	52.6	14	36.8		
Over 6 months	0	0	19	82.6	4	17.4		
Premature							0.417	0.1694
Yes	1	4.3	13	56.5	9	39.1		
No	3	7.9	26	68.4	9	23.7		
Birth weight							0.030*	0.3383
Adequate	4	9.1	31	70.4	9	20.4		
Low weight	0	0	8	47.1	9	52.9		
Pulmonary disease							0.299	0.1990
Yes	0	0	4	100	0	0		
No	4	7.0	35	61.4	18	31.6		
Heart disease							0.201	0.2292
Yes	1	3.7	15	55.6	11	40.7		
No	3	8.8	24	70.6	7	20.6		
Hypothyroidism							0.422	0.1682
Yes	0	0	6	85.7	1	14.3		
No	4	7.4	33	61.1	17	31.5		
Breastfeeding							0.417	0.1694
Yes	4	8.3	29	60.4	15	31.2		
No	0	0	10	76.9	3	23.1		
Bottle feeding							0.124	0.2617
Yes	1	2.4	29	69.0	12	28.6		
No	3	15.8	10	52.6	6	31.6		
Pacifier use							0.552	0.1396
Yes	1	4.7	12	57.1	8	38.1		
No	3	7.5	27	67.5	10	25.0		

A Pearson’s chi-square test;

B Cramer’s V test;

* Significant p-value ≤ 0.05

Caption: n = absolute frequency; % = relative frequency

Table 3 presents the association analysis between habitual tongue posture and the explanatory clinical variables. Tongue posture was associated with breastfeeding; there was a greater number of participants with the predominant tongue posture inside the oral cavity among those who were breastfed, while among those who were not breastfed, there was a greater number of participants with the predominant habitual tongue posture over the lower lip. The effect size for this association was small.

**Table 3:** Association analysis between habitual tongue posture and explanatory clinical variables

Variable	Habitual tongue posture (n = 61)							p-value^A^	Effect size^B^
	Tongue inside the oral cavity			Tongue between gingival ridges		Tongue on lower lip			
	n	%		n	%	n	%		
Sex								0.318	0.1938
Females	11	45.8		7	29.2	6	25.0		
Males	10	27.0		15	40.5	12	32.4		
Age								0.517	0.1471
Up to 6 months	15	39.5		12	31.6	11	28.9		
Over 6 months	6	26.1		10	43.5	7	30.4		
Premature								0.383	0.1775
Yes	6	26.1		8	34.8	9	39.1		
No	15	39.5		14	36.8	9	23.7		
Birth weight								0.842	0.0750
Adequate	16	36.4		15	34.1	13	29.5		
Low weight	5	29.4		7	41.2	5	29.4		
Pulmonary disease								0.192	0.2327
Yes	0	0		3	75.0	1	25.0		
No	21	36.8		19	33.3	17	29.8		
Heart disease								0.572	0.1354
Yes	11	40.7		8	29.6	8	29.6		
No	10	29.4		14	41.2	10	29.4		
Hypothyroidism								0.866	0.0688
Yes	3	42.9		2	28.6	2	28.6		
No	18	33.3		20	37.0	16	29.6		
Breastfeeding								**0.034** ^*^	0.3332
Yes	20	41.7		17	35.4	11	22.9		
No	1	7.7		5	38.5	7	53.8		
Bottle feeding								0.803	0.0849
Yes	15	35.7		14	33.3	13	30.9		
No	6	31.6		8	42.1	5	26.3		
Pacifier use								0.519	0.1465
Yes	7	33.3		6	28.6	8	38.1		
No	14	35.0		16	40.0	10	25.0		

A Pearson’s chi-square test;

B Cramer’s V test;

* Significant p-value ≤ 0.05

Caption: n = absolute frequency; % = relative frequency

## DISCUSSION

The analysis of habitual lip and tongue postures in infants, as presented in this study, reveals associations with clinical variables that may have significant implications for clinical practice and for understanding oral development in infants with T21.

The distribution of habitual lip postures among infants showed a predominance of parted and open lips, with a lower frequency of closed lips. The parted and open lip postures possibly result from hypotonia of the orbicularis oris and levator muscles of the mandible^(18)^, which results in an inability to keep the lips properly closed for long^(4,9)^. Moreover, relative macroglossia, characterized by an increase in the size of the tongue in relation to the oral cavity, can aggravate this condition by occupying more space, becoming protruded, and forcing the lips apart^(8)^. A study^(12)^ found that newborns with a low tongue position in habitual posture kept their lips open/parted, which the authors associated with mouth breathing^(12)^.

This research found an association between lip posture and birth weight. Infants with adequate birth weight presented more favorable lip postures, such as closed or parted lips, than those with low birth weight. This finding is consistent with the literature, which relates birth weight to general motor development^(20)^. No study was found that investigated the influence of weight specifically on the tone and habitual posture of orofacial muscles. However, it is believed that other factors resulting from low weight (e.g., the use of feeding tubes and more frequent hospitalizations in such infants), not investigated in this research, may be associated with this lip posture alteration.

Lip posture was associated with the age of the infants, considering the age of 6 months as the cutoff. It was found that none of the infants older than 6 months had closed lips. The worse posture in older infants observed in this research may reflect a decrease in lip tone with advancing age and/or a progress towards mouth breathing^(11)^. A study with typical infants found a decrease in lip seal with increasing age, concomitant with an increase in oronasal breathing^(21)^. The decrease in lip seal in the aforementioned study occurred around the 8^th^ to 12^th^ month and was associated with respiratory changes and a decrease in breastfeeding^(21)^.

The association analysis between tongue posture and breastfeeding reveals that infants who were breastfed tended to have a more favorable tongue posture, such as the tongue inside the oral cavity, than those who were not breastfed. Infants who are not breastfed may not receive the same oral motor stimulus, resulting in less favorable tongue postures^(22)^. During breastfeeding, the infant's tongue is stimulated to move in a coordinated manner, creating a vacuum for milk extraction^(22)^. Due to the active role of the tongue in sucking at the breast, breastfeeding contributes to this structure’s development^(23)^, which, in turn, can impact its habitual posture.

Inadequate tongue postures (between gingival ridges, on the lower lip, and severe protrusion in relation to the lower lip) accounted for 63.5% of the video duration. This anomalous tongue behavior can be attributed to relative macroglossia and lingual hypotonia, a condition characterized by decreased tongue muscle tone, which makes it difficult to control and maintain correct postures^(11)^. Hypotonia can affect tongue function and lead to inadequate postural compensation, influencing oral health, facial aesthetics, and speech articulation^(8,14)^. This finding agrees with other studies^(4,9)^ that observed a higher incidence of protruding tongue, with improvement in some cases after therapeutic intervention. The literature suggests that the desirable tongue posture (within the oral cavity and elevated) favors longer lip closure time^(12)^ and better palate development^(19)^.

The effect size in these analyses ranged from insignificant to small, with significant associations having small effect sizes. This indicates that, although there is a relationship between the variables, the magnitude of this association is modest – i.e., each associated clinical variable explains only a limited portion of the variability observed in orofacial postures. In clinical practice, this suggests that variables such as age, birth weight, and breastfeeding history do not act alone, but rather in combination with other biological, functional, and/or environmental determinants that influence the orofacial postures of children with T21, reinforcing the multifactorial and complex nature of the orofacial postures adopted by this population.

As limitations of the study, we consider that it only approached clinical variables; there may be other uncontrolled factors that influenced tongue and lip posture, such as socioeconomic factors, maternal health conditions, and complementary feeding practices. It is also worth highlighting the subjective data interpretation of habitual lip and tongue posture, given that the analysis was performed using videos. This limitation was minimized by having two evaluators for the analysis of agreement in 20% of the videos. The study limited the observation time of habitual tongue and lip postures to 5 minutes, for feasibility reasons; however, this period may not have reflected the variability in postures throughout the day or in different situations. Moreover, the absence of sample size calculation reduces statistical robustness and limits the generalization of the results. These limitations should be considered when interpreting the study results and when planning future research that may address these issues more comprehensively.

This study analyzed the association between tongue and lip posture in infants with T21 and important clinical variables, such as sex, age, prematurity, and health conditions, which enriches the understanding of these individuals’ oral motor development. The analysis of intrarater and interrater agreement allowed for greater data reliability. The findings have practical implications for healthcare professionals, such as speech-language-hearing pathologists, in that they highlight the importance of breastfeeding for proper oral development, which can influence recommendations and interventions and reinforce the need for early intervention strategies for children with T21. The results should be interpreted cautiously, as the small convenience sample limits extrapolation. However, they can serve as a basis for future investigations, encouraging studies with larger samples and longitudinal designs to explore more deeply the factors related to the habitual lip and tongue posture of individuals with T21.

## CONCLUSION

The postures adopted for the longest time by infants with T21 were parted lips and tongue inside the oral cavity. Younger chronological age at the time of assessment and higher birth weight were associated with better lip posture, while breastfeeding was associated with better tongue posture.
