# Supplementary material for: Habitual lip and tongue posture in infants with Trisomy 21: characterization and associated factors
Source: Codas. 2026 Jun 19;38(4):e20250157. doi: 10.1590/2317-1782/e20250157en (PMC13322487; doi:10.1590/2317-1782/e20250157en)
Supplement: Translation

# Translation: Postura habitual dos lábios e da língua em lactentes com Trissomia do 21: caracterização e fatores associados

**DOI:** 10.1590/2317-1782/e20250157pt

**Authors:** Letícia Pereira Quintaes Godinho, Larissa Melgaço Campos, Andréa Rodrigues Motta, Renata Maria Moreira Moraes Furlan

**Affiliations:** 1 Universidade Federal de Minas Gerais – UFMG - Belo Horizonte (MG), Brasil.; 2 Programa de Pós-graduação em Ciências Fonoaudiológicas, Universidade Federal de Minas Gerais – UFMG - Belo Horizonte (MG), Brasil.; 3 Departamento de Fonoaudiologia, Universidade Federal de Minas Gerais – UFMG - Belo Horizonte (MG), Brasil.

**Keywords:** Síndrome de Down, Hipotonia Muscular, Cromossomos Humanos Par 21, Língua, Lábio

## Abstract

**Objetivo:**

Caracterizar a postura habitual de lábios e de língua em lactentes com trissomia do 21 e verificar sua associação com idade, sexo e características clínicas.

**Método:**

Trata-se de um estudo observacional, transversal e analítico. A amostra foi composta de 61 lactentes com trissomia do 21, com média de idade de 7,4±6,2 meses. A postura habitual de lábios e de língua foi obtida por meio da análise de vídeos da face dos lactentes. Dados referentes a idade, sexo e condições clínicas foram coletados nos prontuários dos participantes. As variáveis resposta do estudo referem-se às posturas habituais de língua e de lábios predominantemente adotadas pelos lactentes. Constituíram variáveis explicativas: idade, sexo, prematuridade, peso ao nascimento, presença de cardiopatia, hipotireoidismo e doença pulmonar, amamentação e uso de mamadeira e chupeta. Os dados foram analisados por meio de estatística descritiva e teste Qui-quadrado de Pearson para associação entre as variáveis, com nível de significância de 5%. O coeficiente Kappa foi utilizado para obter as concordâncias intra e interavaliador em 20% dos vídeos, sendo classificadas como excelente e substancial, respectivamente.

**Resultados:**

As posturas adotadas por mais tempo foram de lábios semiabertos (48,2%) e de língua dentro da cavidade oral (32,4%). Houve associação da postura de lábios com a idade na data da avaliação e com o peso ao nascimento, e entre a postura de língua e a amamentação.

**Conclusão:**

Menor idade cronológica no momento da avaliação e maior peso ao nascer estiveram associados à melhor postura de lábios, enquanto a presença da amamentação esteve associada à melhor postura de língua.

## INTRODUÇÃO

A trissomia do cromossomo 21 (T21), também conhecida como Síndrome de Down, consiste em uma condição genética causada pela presença de uma cópia extra do cromossomo 21 em todas ou em algumas das células do indivíduo^(1)^. Essa alteração genética resulta em 47 cromossomos, em vez dos 46 habituais, e pode ocorrer por meio de uma divisão celular anormal no momento da fecundação^(2)^. A T21 implica em características físicas e cognitivas, como o atraso no desenvolvimento motor e intelectual, e peculiaridades faciais, como olhos em formato de amêndoa, pescoço curto e hipotonia muscular^(1)^. A síndrome afeta aproximadamente 1 em 800 nascidos vivos, sendo uma das condições genéticas mais comuns^(2)^. A incidência dessa condição aumenta com a idade materna avançada^(3)^.

Crianças com T21 frequentemente apresentam alterações fonoaudiológicas que envolvem tanto a motricidade orofacial quanto a comunicação^(4,5)^. Essas crianças podem demonstrar dificuldades significativas na alimentação, incluindo problemas na amamentação^(6)^ e na deglutição^(7)^. Além disso, o atraso no desenvolvimento da linguagem é uma característica comum, bem como as dificuldades no desenvolvimento da fala e na articulação das palavras^(5)^. A presença de alterações anatômicas, como a macroglossia e o palato alto e estreito, podem agravar essas dificuldades^(8)^.

Estudos que avaliaram as estruturas orofaciais de lactentes com T21 encontraram lábios e língua hipotônicos, impactando na postura habitual dessas estruturas, sugerindo que a diminuição do tônus nos músculos labiais e mandibulares leva à dificuldade em manter os lábios fechados, resultando em posturas de lábios semiabertos ou abertos^(4,9)^. Em indivíduos com T21 a hipotonia muscular afeta toda a musculatura corporal de maneira generalizada, sendo explicada por características estruturais e funcionais do sistema nervoso central, como redução do volume e número de células do córtex cerebral e do cerebelo e alteração da conectividade entre os neurônios e demais estruturas^(10)^.

A alteração na posição habitual dos lábios e da língua tem relação com o modo respiratório, sendo verificadas posturas de lábios entreabertos e língua em assoalho da boca em crianças respiradoras orais^(11)^. Um estudo^(12)^ observou que a postura da língua dentro da cavidade oral e em posição mais elevada, em recém-nascidos típicos, esteve associada a um fluxo expiratório mais simétrico, sugerindo que a relação entre a respiração e a postura da língua pode ser encontrada já nos primeiros dias de vida. Isso reforça a importância de um olhar direcionado às posturas habituais orofaciais de lactentes, dados os prejuízos da respiração oral para o desenvolvimento infantil e qualidade de vida^(13)^. Além disso, a presença de posturas inadequadas dessas estruturas pode impactar negativamente no desenvolvimento craniofacial, com possíveis consequências a longo prazo, como por exemplo, hipodesenvolvimento da maxila e más-oclusões dentárias^(8,14)^.

Embora estudos abordem as alterações miofuncionais orofaciais em crianças com T21, apontando a presença de posturas inadequadas de lábios e de língua^(4,9)^, não foram encontrados estudos que investigassem os fatores associados à adoção dessas posturas, ademais ainda não se sabe com clareza quais fatores podem influenciar a postura habitual da língua e dos lábios nessas crianças. Compreender tais fatores é essencial, pois permitiria um diagnóstico mais preciso e uma abordagem melhor direcionada na intervenção, a fim de minimizar os impactos negativos das alterações posturais e, por consequência, garantir um desenvolvimento orofacial saudável.

Diante disso, o objetivo do presente estudo é caracterizar a postura habitual de lábios e de língua em lactentes com trissomia do cromossomo 21 e verificar a associação com idade, sexo e características clínicas.

## MÉTODO

Este estudo adotou uma abordagem quantitativa e seguiu um desenho observacional transversal e analítico. O estudo foi aprovado pelo Comitê de Ética em Pesquisa da Universidade Federal de Minas Gerais (UFMG) sob o parecer 4.381.966, CAAE 37828920.1.0000.5149. Todos os pais e/ou responsáveis pelos lactentes assinaram o Termo de Consentimento Livre e Esclarecido.

A coleta de dados ocorreu na Faculdade de Odontologia da UFMG e envolveu 61 lactentes participantes de um projeto de extensão destinado a lactentes com T21. Todos os pacientes do projeto que atenderam aos critérios estabelecidos foram incluídos no estudo. Não foi realizado o cálculo amostral; a amostra foi determinada com base no número de bebês atendidos, conforme banco de dados do referido projeto de extensão.

Foram incluídos os lactentes com idade gestacional corrigida entre 6 meses e 3 anos, com diagnóstico de T21. Foram excluídos lactentes com outras síndromes associadas, más-formações orofaciais, ou utilização de sondas enterais. A verificação dos critérios de exclusão foi realizada mediante análise dos prontuários dos lactentes do projeto de extensão.

A fim de atender os objetivos propostos, o estudo contou com duas etapas: coleta de dados clínicos pela análise dos prontuários dos participantes e acesso à gravação de vídeos da face dos lactentes para observação da postura habitual de lábios e de língua.

### Histórico clínico

Foram extraídas dos prontuários informações sobre a idade, sexo e características clínicas dos lactentes; incluindo histórico de saúde (síndromes associadas, más-formações, prematuridade, peso ao nascer, presença de doença pulmonar, cardiopatia, hipotireoidismo), presença de aleitamento materno, uso de sonda de alimentação, mamadeira e chupeta.

### Análise da postura habitual de lábios e de língua

A postura habitual dos lábios e da língua foi verificada por meio da análise de vídeos da face dos lactentes, habitualmente realizados na primeira consulta no projeto de extensão. Para a gravação os lactentes foram posicionados no colo dos pais, os quais se encontravam sentados em uma cadeira com encosto. Os vídeos foram focados na face do lactente e tiveram duração de 5 minutos.

Os vídeos foram analisados, no *software* Media Player Classic^®^, sendo pausados segundo a segundo. Em cada segundo, a postura de língua foi classificada como: I) dentro da cavidade oral (língua atrás do rolete gengival inferior ou atrás dos dentes incisivos inferiores); II) entre os roletes gengivais (língua sobre o rolete gengival inferior e atrás do lábio inferior); III) sobre o lábio inferior (língua tocando o lábio inferior); IV) protrusão severa em relação ao lábio inferior (língua protruída, sobre o lábio inferior, com o ápice ultrapassando o limite anterior do lábio inferior)^(4)^. A postura de lábios foi classificada como: I) fechados (presença de contato entre os lábios inferior e superior em toda a extensão da rima labial); II) semiabertos (contato entre os lábios superior e inferior apenas próximo às comissuras labiais); ou III) abertos (não há contato entre lábio inferior e superior)^(4)^. Foram contabilizados os segundos em que o lactente permaneceu em cada classificação da postura habitual de lábios e de língua, sendo considerada como predominante a postura adotada durante a maior parte do tempo de vídeo, na comparação com as demais posturas isoladas. Os momentos em que o lactente sorriu ou vocalizou não foram considerados na análise. Uma pesquisadora analisou todos os vídeos e, para aumentar a confiabilidade dos dados, uma segunda pesquisadora realizou a análise de 20% dos vídeos de forma independente, sendo verificada a concordância interavaliador. Além disso, a primeira pesquisadora reanalisou 20% dos vídeos, após 6 meses da primeira análise, sendo verificada a concordância intra-avaliador. Para verificação das concordâncias inter e intra-avaliador, foi calculado o coeficiente Kappa^(15)^.

### Metodologia de análise dos dados

As variáveis resposta do estudo foram: postura habitual de lábios e língua predominantemente adotadas pelos lactentes; já as variáveis explicativas foram: sexo, idade, prematuridade, peso ao nascer, doença pulmonar, cardiopatia, hipotireoidismo, amamentação, mamadeira e chupeta.

Foi realizada a análise estatística descritiva, com apresentação de medidas de tendência central e dispersão para as variáveis numéricas: porcentagem de tempo nas posturas de lábios e de língua e idade na data da avaliação. Variáveis categóricas (sexo, prematuridade, doença pulmonar, cardiopatia, hipotireoidismo, amamentação, uso de mamadeira e chupeta) foram descritas por meio das frequências absolutas e relativas. As variáveis numéricas, idade na data da avaliação e peso ao nascimento foram transformadas em categóricas, considerando o ponto de corte de seis meses para a idade na data da avaliação e de 2.500 g como o ponto de corte do peso ao nascimento (baixo peso: abaixo de 2.500 g; adequado: igual ou acima de 2.500 g)^(16)^.

A análise estatística inferencial foi realizada por meio do teste Qui-quadrado de Pearson para associação entre as variáveis. Adotou-se um nível de significância de 5% em todas as análises. Utilizou-se o software Stata 13.0.

O tamanho do efeito foi calculado, sendo considerado insignificante quando menor que 0,19; pequeno entre 0,20 e 0,49; médio para valores entre 0,50 e 0,79 e grande quando maior que 0,80^(17)^.

Para análise de concordância foi realizada a comparação interavaliador e intra-avaliador (Tabela 1). A concordância interavaliador foi classificada como substancial tanto para lábios quanto para língua, enquanto a concordância intra-avaliador foi excelente para ambas as posturas, de acordo com os critérios de Landis e Koch^(15)^.

**Tabela 1:** Resultado da concordância interavaliador e intra-avaliador

Variável	Concordância Interavaliador		Concordância Intra-avaliador	
	kappa	Classificação	kappa	Classificação
Postura habitual de lábios	0,7477	Substancial	0,815	Excelente
Postura habitual de língua	0,7544	Substancial	0,900	Excelente

Teste Kappa

Legenda: Classificação: fraca (0 a 0,2); razoável (0,21 a 0,40); moderada (0,41 a 0,60); substancial (0,61 a 0,80); e excelente (0,81 a 1,0)^(15)^

## RESULTADOS

Participaram da pesquisa 61 lactentes, com média de idade, na data da avaliação, de 7,4 meses (DP=6,2 meses, mínimo de 1 mês e máximo de 26 meses), sendo 24 do sexo feminino (39,3%) e 37 do sexo masculino (60,7%).

As Figuras 1 e 2 apresentam a quantidade de tempo, em média, que os lactentes assumiram cada uma das posturas habituais de lábios e de língua, respectivamente, apresentadas em porcentagem da duração máxima dos vídeos. As posturas adotadas por mais tempo foram a de lábios semiabertos e a de língua dentro da cavidade oral, nos vídeos analisados. As posturas inadequadas da língua combinadas somam 63,5% do tempo dos vídeos e as de lábios 80,2%.

**Figura 1:** Duração de cada postura habitual de lábios em porcentagem do tempo total dos vídeos

**Figura 2:** Duração de cada postura habitual de língua em porcentagem do tempo total dos vídeos

Com relação ao número de indivíduos classificados de acordo com a sua postura habitual predominante, houve maior frequência de indivíduos com predominância de lábios semiabertos (n=39, 63,9%), seguida de lábios abertos (n=18, 29,6%) e de lábios fechados (n=4, 6,5%). Houve maior número de indivíduos classificados com postura predominante de língua entre os roletes gengivais (n=22, 36,1%), seguida de dentro da cavidade oral (n=21, 34,4%) e sobre o lábio inferior (n=18, 29,5%). Nenhum participante apresentou a protrusão severa como postura de língua predominante.

A Tabela 2 apresenta a análise de associação entre a postura habitual de lábios e as variáveis clínicas explicativas. Houve associação entre a postura de lábios e o peso ao nascimento, com maior quantidade de participantes que nasceram com peso adequado apresentando predominantemente postura de lábios semiabertos e maior quantidade de lactentes que nasceram com baixo peso apresentando predominantemente lábios abertos. Também houve associação entre a postura de lábios e a idade na data da avaliação, sendo que nenhum dos participantes avaliados após 6 meses apresentaram lábios fechados como postura predominante. O tamanho do efeito foi pequeno para ambas as associações encontradas.

**Tabela 2:** Análise de associação postura habitual de lábios e as variáveis clínicas explicativas

Variável	Postura habitual de lábios (n=61)						Valor de p^A^	Tamanho do Efeito^B^
	Lábios fechados		Lábios semiabertos		Lábios abertos			
	n	%	n	%	n	%		
Sexo							0,822	0,0802
Feminino	1	4,2	16	66,7	7	29,2		
Masculino	3	8,1	23	62,2	11	29,7		
Idade							0,043*	0,3207
Até 6 meses	4	10,5	20	52,6	14	36,8		
Após 6 meses	0	0	19	82,6	4	17,4		
Prematuridade							0,417	0,1694
Sim	1	4,3	13	56,5	9	39,1		
Não	3	7,9	26	68,4	9	23,7		
Peso ao nascer							**0,030** ^*^	0,3383
Adequado	4	9,1	31	70,4	9	20,4		
Baixo Peso	0	0	8	47,1	9	52,9		
Doença pulmonar							0,299	0,1990
Sim	0	0	4	100	0	0		
Não	4	7,0	35	61,4	18	31,6		
Cardiopatia							0,201	0,2292
Sim	1	3,7	15	55,6	11	40,7		
Não	3	8,8	24	70,6	7	20,6		
Hipotireoidismo							0,422	0,1682
Sim	0	0	6	85,7	1	14,3		
Não	4	7,4	33	61,1	17	31,5		
Amamentação							0,417	0,1694
Sim	4	8,3	29	60,4	15	31,2		
Não	0	0	10	76,9	3	23,1		
Mamadeira							0,124	0,2617
Sim	1	2,4	29	69,0	12	28,6		
Não	3	15,8	10	52,6	6	31,6		
Chupeta							0,552	0,1396
Sim	1	4,7	12	57,1	8	38,1		
Não	3	7,5	27	67,5	10	25,0		

A Teste Qui-quadrado de Pearson;

B Teste V de Cramér;

* Valor de p significante ≤0,05

Legenda: n = frequência absoluta; % = frequência relativa

A Tabela 3 apresenta a análise de associação entre a postura habitual de língua e as variáveis clínicas explicativas. Houve associação entre a postura de língua e a amamentação, com maior quantidade de participantes com a postura de língua predominante dentro da cavidade oral entre os que foram amamentados, enquanto entre os que não foram amamentados houve maior número de participantes com postura habitual de língua predominante sobre o lábio inferior, sendo o tamanho do efeito pequeno para esta associação.

**Tabela 3:** Análise de associação postura habitual de língua e as variáveis clínicas explicativas

Variável	Postura habitual de língua (n=61)						Valor de p^A^	Tamanho do Efeito^B^
	Língua dentro da cavidade oral		Língua entre os roletes gengivais		Língua sobre o lábio inferior			
	n	%	n	%	n	%		
Sexo							0,318	0,1938
Feminino	11	45,8	7	29,2	6	25,0		
Masculino	10	27,0	15	40,5	12	32,4		
Idade							0,517	0,1471
Até 6 meses	15	39,5	12	31,6	11	28,9		
Após 6 meses	6	26,1	10	43,5	7	30,4		
Prematuridade							0,383	0,1775
Sim	6	26,1	8	34,8	9	39,1		
Não	15	39,5	14	36,8	9	23,7		
Peso ao nascer							0,842	0,0750
Adequado	16	36,4	15	34,1	13	29,5		
Baixo Peso	5	29,4	7	41,2	5	29,4		
Doença Pulmonar							0,192	0,2327
Sim	0	0	3	75,0	1	25,0		
Não	21	36,8	19	33,3	17	29,8		
Cardiopatia							0,572	0,1354
Sim	11	40,7	8	29,6	8	29,6		
Não	10	29,4	14	41,2	10	29,4		
Hipotireoidismo							0,866	0,0688
Sim	3	42,9	2	28,6	2	28,6		
Não	18	33,3	20	37,0	16	29,6		
Amamentação							**0,034** ^*^	0,3332
Sim	20	41,7	17	35,4	11	22,9		
Não	1	7,7	5	38,5	7	53,8		
Mamadeira							0,803	0,0849
Sim	15	35,7	14	33,3	13	30,9		
Não	6	31,6	8	42,1	5	26,3		
Chupeta							0,519	0,1465
Sim	7	33,3	6	28,6	8	38,1		
Não	14	35,0	16	40,0	10	25,0		

A Teste Qui-quadrado de Pearson;

B Teste V de Cramér;

* Valor de p significante ≤0,05

Legenda: n = frequência absoluta; % = frequência relativa

## DISCUSSÃO

A análise das posturas habituais de lábios e de língua em lactentes, conforme apresentado neste estudo, revela associações com variáveis clínicas que podem ter implicações significativas para a prática clínica e para a compreensão do desenvolvimento oral em lactentes com T21.

A distribuição das posturas habituais de lábios entre os lactentes evidenciou uma predominância de lábios semiabertos e abertos, com menor frequência de lábios fechados. A postura de lábios semiabertos e abertos possivelmente decorre da hipotonia dos músculos orbicular e levantadores da mandíbula^(18)^, o que resulta em uma incapacidade de manter os lábios fechados adequadamente por um longo período de tempo^(4,9)^. Além disso, a macroglossia relativa, caracterizada pelo aumento do tamanho da língua em relação à cavidade oral, pode agravar essa condição ao ocupar mais espaço, ficar protruída e forçar os lábios a permanecerem afastados^(8)^. Um estudo^(12)^ constatou que recém-nascidos que apresentaram posição de língua baixa em postura habitual mantiveram os lábios abertos/entreabertos, o que foi associado pelos autores com o modo respiratório oral^(12)^.

Verificou-se, nesta pesquisa, associação entre a postura labial e o peso ao nascer. Lactentes com peso adequado ao nascer apresentaram posturas labiais mais favoráveis, como lábios fechados ou semiabertos, em comparação com aqueles com baixo peso. Esse achado é concordante com a literatura, que relaciona peso ao nascer ao desenvolvimento motor geral^(20)^. Não foi encontrado um estudo que investigasse a influência do peso especificamente no tônus e na postura habitual de músculos orofaciais. No entanto, acredita-se que outros fatores decorrentes do baixo peso, não investigados nesta pesquisa, podem estar associados a essa alteração postural nos lábios, por exemplo, uso de sondas de alimentação e internações mais frequentes nesses lactentes.

A postura dos lábios apresentou associação com a idade dos lactentes, considerando a idade de 6 meses como ponto de corte. Constatou-se que nenhum dos lactentes maiores de seis meses apresentava lábios fechados. A pior postura, observada nesta pesquisa, nos lactentes mais velhos pode refletir uma diminuição do tônus labial com o avançar da idade e/ou uma evolução para um modo respiratório oral^(11)^. Um estudo realizado com lactentes típicos verificou diminuição do vedamento labial com o aumento da idade, concomitante ao aumento da respiração oronasal^(21)^. A diminuição do vedamento labial no referido estudo ocorreu em torno do 8º ao 12º mês e esteve associada a alterações respiratórias e diminuição do aleitamento materno^(21)^.

A análise da associação entre a postura de língua e a amamentação revela que lactentes que foram amamentados tenderam a apresentar postura de língua mais favorável, como a língua dentro da cavidade oral, em comparação com aqueles que não foram amamentados. Lactentes que não são amamentados podem não receber o mesmo estímulo motor oral, resultando em posturas de língua menos favoráveis^(22)^. Durante a amamentação, a língua do lactente é estimulada a se mover de maneira coordenada, criando um vácuo para a extração do leite^(22)^. Devido ao papel ativo da língua na sucção ao seio, a amamentação contribui no processo de desenvolvimento dessa estrutura^(23)^, o que, por sua vez, pode impactar na sua postura habitual.

As posturas inadequadas de língua (entre roletes gengivais, sobre lábio inferior e protrusão severa em relação ao lábio inferior) corresponderam a 63,5% da duração dos vídeos. Esse comportamento anômalo da língua pode ser atribuído à macroglossia relativa e à hipotonia lingual, condição caracterizada pela diminuição do tônus muscular da língua, o que dificulta o controle e a manutenção de posturas corretas^(11)^. A hipotonia pode afetar a função lingual e levar a compensações posturais inadequadas, influenciando tanto a saúde oral quanto a estética facial e a articulação da fala^(8,14)^. Este achado concorda com outros estudos^(4,9)^ que observaram maior incidência de língua protrusa, com melhora em alguns casos após a intervenção terapêutica. A literatura sugere que a postura desejável da língua, dentro da cavidade oral e elevada, favorece maior tempo de vedamento labial^(12)^ e melhor desenvolvimento do palato^(19)^.

O tamanho de efeito, nessas análises, variou de insignificante a pequeno, sendo que as associações significativas tiveram tamanho de efeito pequeno. Isso indica que, apesar de existir relação entre as variáveis, a magnitude dessa associação é modesta, ou seja, cada variável clínica associada explica apenas uma parcela limitada da variabilidade observada nas posturas orofaciais. Na prática clínica, isso sugere que variáveis como idade, peso ao nascer e histórico de amamentação não atuam de forma isolada, mas sim em conjunto com outros determinantes biológicos, funcionais e/ou ambientais que influenciam nas posturas orofaciais de crianças com T21, reforçando a natureza multifatorial e complexa das posturas orofaciais adotadas por essa população.

Como limitações do estudo, considera-se o fato de se ter considerado apenas variáveis clínicas, podendo haver outros fatores não controlados que influenciaram a postura da língua e dos lábios, como fatores socioeconômicos, condições de saúde materna e práticas de alimentação complementares. Ressalta-se ainda a subjetividade na interpretação dos dados da postura habitual de lábios e de língua, tendo em vista que a análise foi realizada por meio de vídeos. Nesse sentido, a presença de duas avaliadoras para análise de concordância de 20% dos vídeos foi adotada para minimizar essa limitação. O estudo limitou o tempo de observação das posturas habituais de língua e de lábios em 5 minutos, por questões de viabilidade, sendo que o período pode não ter refletido a variabilidade nas posturas ao longo do dia ou em diferentes situações. Além disso, a ausência de cálculo amostral reduz a robustez estatística e limita a generalização dos resultados. Essas limitações devem ser consideradas ao interpretar os resultados do estudo e ao planejar pesquisas futuras que possam abordar essas questões de forma mais abrangente.

Este estudo analisou a associação entre a postura da língua e dos lábios em lactentes com T21 e variáveis clínicas importantes, como sexo, idade, prematuridade e condições de saúde, o que enriquece a compreensão do desenvolvimento motor oral desses indivíduos. A análise das concordâncias intra e interavaliador possibilitou uma maior confiabilidade dos dados. Os achados têm implicações práticas para profissionais de saúde, como fonoaudiólogos, ao destacar a importância da amamentação para o desenvolvimento oral adequado, o que pode influenciar recomendações e intervenções, e reforçam a necessidade de estratégias de intervenção precoce em crianças com T21. Os resultados devem ser interpretados com cautela, visto que a amostra reduzida e por conveniência limita a extrapolação. Porém, podem servir como base para futuras investigações, incentivando estudos com amostras mais amplas e delineamento longitudinal, que possam explorar mais profundamente os fatores relacionados à postura habitual de lábios e de língua de indivíduos com T21.

## CONCLUSÃO

As posturas adotadas por mais tempo pelos lactentes com trissomia do cromossomo 21 foram de lábios semiabertos e de língua dentro da cavidade oral. A menor idade cronológica na data da avaliação e o maior peso ao nascer estiveram associados à melhor postura dos lábios, enquanto a presença da amamentação esteve associada à melhor postura de língua.
